# Unsupervised class discovery in pancreatic ductal adenocarcinoma reveals cell-intrinsic mesenchymal features and high concordance between existing classification systems

**DOI:** 10.1038/s41598-019-56826-9

**Published:** 2020-01-15

**Authors:** Frederike Dijk, Veronique L. Veenstra, Eline C. Soer, Mark P. G. Dings, Lan Zhao, Johannes B. Halfwerk, Gerrit K. Hooijer, Helene Damhofer, Marco Marzano, Anne Steins, Cynthia Waasdorp, Olivier R. Busch, Marc G. Besselink, Johanna A. Tol, Lieke Welling, Lennart B. van Rijssen, Sjors Klompmaker, Hanneke W. Wilmink, Hanneke W. van Laarhoven, Jan Paul Medema, Louis Vermeulen, Sander R. van Hooff, Jan Koster, Joanne Verheij, Marc J. van de Vijver, Xin Wang, Maarten F. Bijlsma

**Affiliations:** 1 Department of Pathology, Amsterdam UMC, University of Amsterdam and Cancer Center Amsterdam, Amsterdam, Netherlands; 2 Laboratory for Experimental Oncology and Radiobiology, Amsterdam UMC, University of Amsterdam and Cancer Center Amsterdam, Amsterdam, Netherlands; 3 Oncode Institute, Amsterdam, the Netherlands; 4 Department of Biomedical Sciences, City University of Hong Kong, Kowloon Tong, Hong Kong; 5 Present Address: Cell Biology Program, Memorial Sloan Kettering Cancer Center, New York, United States of America; 6 Department of Surgery, Amsterdam UMC, University of Amsterdam and Cancer Center Amsterdam, Amsterdam, Netherlands; 7 Present Address: Department of Surgery, Leiden University Medical Centre, Leiden, The Netherlands; 8 Department of Medical Oncology, Amsterdam UMC, University of Amsterdam and Cancer Center Amsterdam, Amsterdam, Netherlands; 9 Department of Oncogenomics, Amsterdam UMC, University of Amsterdam and Cancer Center Amsterdam, Amsterdam, Netherlands; 10 Shenzhen Research Institute, City University of Hong Kong, Shenzhen, China

**Keywords:** RNA sequencing, Pancreatic cancer, Tumour heterogeneity, Functional clustering

## Abstract

Pancreatic ductal adenocarcinoma (PDAC) has the worst prognosis of all common cancers. However, divergent outcomes exist between patients, suggesting distinct underlying tumor biology. Here, we delineated this heterogeneity, compared interconnectivity between classification systems, and experimentally addressed the tumor biology that drives poor outcome. RNA-sequencing of 90 resected specimens and unsupervised classification revealed four subgroups associated with distinct outcomes. The worst-prognosis subtype was characterized by mesenchymal gene signatures. Comparative (network) analysis showed high interconnectivity with previously identified classification schemes and high robustness of the mesenchymal subtype. From species-specific transcript analysis of matching patient-derived xenografts we constructed dedicated classifiers for experimental models. Detailed assessments of tumor growth in subtyped experimental models revealed that a highly invasive growth pattern of mesenchymal subtype tumor cells is responsible for its poor outcome. Concluding, by developing a classification system tailored to experimental models, we have uncovered subtype-specific biology that should be further explored to improve treatment of a group of PDAC patients that currently has little therapeutic benefit from surgical treatment.

## Introduction

Pancreatic ductal adenocarcinoma (PDAC) is the most lethal of all common solid tumors with reported 5-year survival rates below 8%^1^. This poor prognosis can be largely attributed to diagnosis at late disease stage, when surgical resection is no longer possible. In patients eligible for surgery, systemic chemotherapy, like gemcitabine and more recently FOLFIRINOX, only marginally prolongs survival^2–4^.

Clinical trials have typically failed in unselected PDAC cohorts, demonstrating that patient classification is essential for the efficacy of novel therapeutic approaches^5^. The expectation was that mutational profiling would better identify surgical candidates or predict favorable responses to (neo)adjuvant or palliative systemic treatment^6^. Although this approach identified many genetic alterations in PDAC^7–11^, clinical applicability remains limited^12^. Instead, capturing the intertumor heterogeneity that underlies poor outcomes or responsiveness is arguably best achieved by transcriptome analysis. Transcriptomic analyses capture both the intrinsic and extrinsic factors that drive tumor cell growth. We speculate that the interplay between these two variables drives the response to treatment. For pancreatic cancer, gene expression profiling and supervised class discovery studies have been performed^13–19^. In addition, effective unsupervised RNA-based classification studies, also in PDAC, have been completed^20–27^. In spite of the proposed molecular classifications thus far, the cellular and molecular mechanisms that cause the observed disease outcomes or therapeutic responses remain largely unknown. We still lack critical information to study the underlying disease-relevant mechanisms, which will allow the development of much needed new treatments.

To address this need, we first performed unsupervised classification on a unique single-center single-platform set of histopathologically revised PDAC-only samples from patients who underwent surgery. This revealed the existence of subgroups of PDAC with highly divergent outcomes, which bear high similarity to previously identified molecular subtypes as revealed by network analysis comparisons. Next, using these data and matching patient-derived xenografts, we established a molecular classification of experimental models for PDAC by constructing dedicated PDX- and cell line-optimized classifiers. We were able to experimentally demonstrate that the worst-outcome subgroup is defined by highly invasive tumor growth and that these mesenchymal features are tumor cell-intrinsic. The ability to identify those patients that will likely not benefit from surgery alone will dramatically aid clinical decision-making. In addition, the identification of experimental models for mesenchymal PDAC could be used to reveal subtype-specific vulnerabilities that could ultimately increase survival rates of those patients that do not benefit from resection of the tumor alone.

## Results

### Unsupervised class discovery in PDAC reveals four distinct subgroups

**Figure 1:**
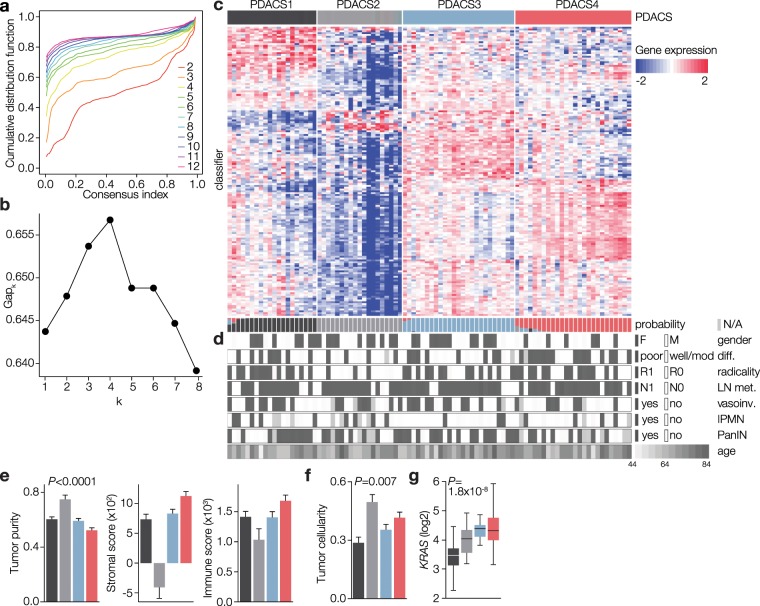
Unsupervised class discovery in PDAC reveals four distinct subgroups. (**a)** Unsupervised consensus clustering was performed and four clusters were identified. Cumulative distribution function (CDF) is shown for cluster numbers k = 2–12 (indicated with coloured lines). (**b**) Stability of identified cluster numbers (range indicated on x-axis) was assessed by GAP statistics for k = 1–8, 8050 genes with average log2 (RPKM) > 1 and median absolute deviation > 0.5 across samples. The optimal cluster number was found to be four. (**c**) A classifier was constructed and patients were assigned to the different PDACS subgroups shown in the top bar. Heatmap shows the cluster analysis with 159 signature genes in rows. Bottom bars indicate posterior probability of the subtype indicated by corresponding colours. (**d**) Association of PDACS subgroups with clinical variables. Differentiation grade grouped in poor vs well/moderately differentiated. See also Table 1. (**e**) ESTIMATE-derived tumor purity, stromal content and immune score for the four subtypes indicated by bar colors. *P* < 0.0001 is significance for all three graphs, ANOVA-tested. (**f**) Pathologist-scored tumor cellularity shown per subtype. Significance was determined by Chi-square (scored cellularities are categorical). (**g**) *KRAS* transcript levels shown per PDACS group. Significance was tested by ANOVA.

Previous classification studies in pancreatic cancer identified clinically relevant subtypes. However, functional assessment of the tumor biology that underlies subtype-specific outcomes in experimental models has lagged behind. This critical information should lead to more effective patient stratification and development of new therapeutic targets. To address this, we first assembled a large, unique single-center single-platform PDAC-only set with matching model systems. We selected tumor specimens from 230 retrospectively and 115 prospectively collected samples. After assessing tumor cellularity and histopathological confirmation of PDAC diagnosis, 90 samples were found to be appropriate for RNA-Seq. Following RNA-Seq, we performed unsupervised consensus clustering and determined the optimal number of clusters to be four (Fig. 1a,b and Supplementary Fig. 1). We then established a 159-gene classifier using PAM^28^ (Supplementary Table 1) and classified approximately equal patient numbers to the four PDAC subtypes (PDACS; Fig. 1c).

**Figure 2:**
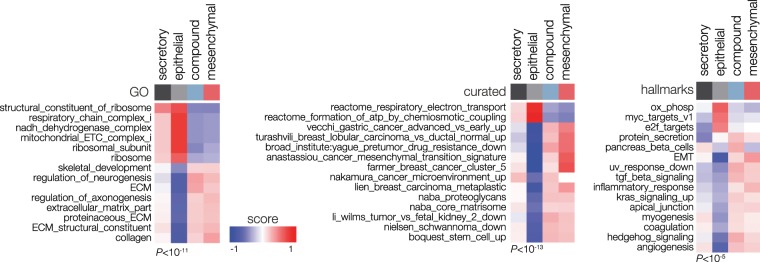
PDACS groups associate with distinct biological programs. Gene set analysis using the MSigDB collections; hallmarks (collection H), gene ontology (GO; C5), and curated (C2). Significance threshold is indicated below heatmaps. Colours indicate gene set Z-score. Coloured bars on top indicate subtypes.

Gene set analysis revealed that each of the distinct PDAC subtypes was characterized by specific biological features (Fig. 2 and Supplementary Fig. 2a). Based on these analyses, we named the identified subtypes *secretory*, *epithelial*, *compound pancreatic* and *mesenchymal*. The secretory subtype was enriched for endocrine and exocrine functions of the pancreas, as indicated by Moffitt’s endocrine and exocrine factors and β-cell and pancreatic secretion signatures (Fig. 2 and Supplementary Fig. 2). As neuroendocrine transdifferentiation has recently been associated with poor outcome in PDAC^29^, these enrichments could indicate high lineage plasticity. Epithelial subtype tumors were characterized by Myc signaling, high expression of mitochondrial components and ribosome signatures. The mesenchymal subtype was enriched for signatures for epithelial-to-mesenchymal transition, and stroma and TGF-β signaling. The compound pancreatic subtype featured signatures similar to the mesenchymal subtype but with endocrine characteristics.

### PDACS groups associate with outcome

Overall, clinical parameters, including age and histologic type, were similar among the subtypes (Fig. 1d and Table 1). Targeted mutation analysis for common PDAC driver genes (*TP53*, *SMAD4*, *KRAS*) revealed no correlation to the PDACS groups (*P* = 0.52; *P* = 0.15; *P* = 0.58 respectively, not shown in table). However, we found significant differences between radicality of resection (i.e. whether the resection margins were free of tumor) and tumor cell percentage. Epithelial subtype-patients more often featured radical resections (R0; Pearson χ^2^ test *P* = 0.013). Assessments of tumor purity and stromal and immune cell content by ESTIMATE^30^ revealed that the epithelial subtype (PDACS2) was relatively enriched in tumor cells (Fig. 1e). We confirmed this finding by histopathological assessment of tumor cellularity (Fig. 1f and Table 1). We also found that the mesenchymal subtype had the highest *KRAS* transcript levels (Fig. 1g), which is in line with recent work showing that mesenchymal, high grade PDAC, features genetic gains in oncogenic *KRAS*^31^.

**Table 1:** Clinicopathological characteristics of the PDACS groups.

Patients		*N*	(%)	PDACS1	PDACS2	PDACS3	PDACS4	*P*-value
		90		*N* = 20	*N* = 19	*N* = 25	*N* = 26	
Sex	male	54	(60.0)	12	9	13	20	0.17
	female	36	(40.0)	8	10	12	6	
	*ratio*	*1.5*		*1.5*	*0.9*	*1.1*	*3.3*	
Age	median	66		65	62	67	70	0.44
	*range*	*(44–83)*		*(44–76)*	*(53–83)*	*(52–81)*	*(48–80)*	
Type of resection	PPPD	79	(87.8)	17	16	22	24	0.52
	Whipple	8	(8.9)	2	3	1	2	
	Corpus/tail res.	3	(3.3)	1		2		
Tumor cell percentage	median	35.0		32.5	50	35	37.5	**0.007**
	*range*	*(10–80)*		*(10–70)*	*(10–70)*	*(10–75)*	*(10–80)*	
RIN	median	8.0		6.9	7.6	8.8	8	0.23
	*range*	*(3.2–9.7)*		*(3.2–9.7)*	*(6.0–9.6)*	*(6.2–9.4)*	*(6.8–8.9)*	
Diameter of tumor (cm)	median	3.0		2.6	3.5	2.8	3	0.81
	*range*	*(0.8–8.5)*		*(0.8–8.5)*	*(2.0–6.5)*	*(1.7–5.2)*	*(1.5–4.5)*	
	unknown	1	(1.1)			1		
Histologic type	pancreaticobiliary	77	(85.5)	18	15	22	22	0.24
	intestinal	7	(7.8)	1	4	1	1	
	unknown	6	(6.7)	1		2	3	
Radicality	R0	47	(52.2)	7	16	12	12	**0.01**
	R1	43	(47.8)	13	3	13	14	
	*ratio*		*1.1*					
Differentiation grade	well	8	(8.9)	4	1	3		0.15
	moderate	44	(48.9)	8	13	13	10	
	poor	35	(38.9)	8	4	8	15	
	unknown	3	(3.3)		1	1	1	
Perineural growth	no	20	(22.2)	3	5	3	9	0.42
	yes	64	(71.1)	15	12	21	16	
	unknown	6	(6.7)	2	2	1	1	
Vasoinvasive growth	no	50	(55.6)	11	9	13	17	0.41
	yes	34	(37.8)	9	7	11	7	
	unknown	6	(6.7)		3	1	2	
IPMN component	no	64	(71.1)	12	11	22	19	0.25
	yes	16	(17.8)	5	4	3	4	
	unknown	10	(11.1)	3	4		3	
PanIN component	no	37	(41.1)	8	6	10	13	0.18
	yes	44	(48.9)	11	9	15	9	
	unknown	9	(10.0)	1	4		4	
Lymph node	no	20	(22.2)	6	4	5	5	0.82
metastasis	yes	70	(77.8)	14	15	20	21	
Distant	no	87	(98.9)	19	18	24	26	0.7
metastasis	yes	3	(1.1)	1	1	1		
Neoadjuvant therapy	none	86	(95.6)	17	19	24	26	0.15
	FOLFIRINOX	2	(2.2)	2		1		
	gem + radioth.	2	(2.2)	1				
Adjuvant	none	57	(63.3)	11	12	16	18	0.80
therapy	gemcitabine	33	(36.7)	9	7	9	8	
Status	alive	9		1	4	3	1	
	dead	81		19	15	22	25	
OS	median (months)	17.5		15.7	29.2	21.5	14.5	0.46
	*range*	*(0.5–12)*		*(2.2–52.5)*	*(2–114.5)*	*(5.8–95.4)*	*(5.5–57.0)*	

Bold lettering indicates significance p < 0.05. Italics indicate data ranges or ratios calculated from data shown.

**Figure 3:**
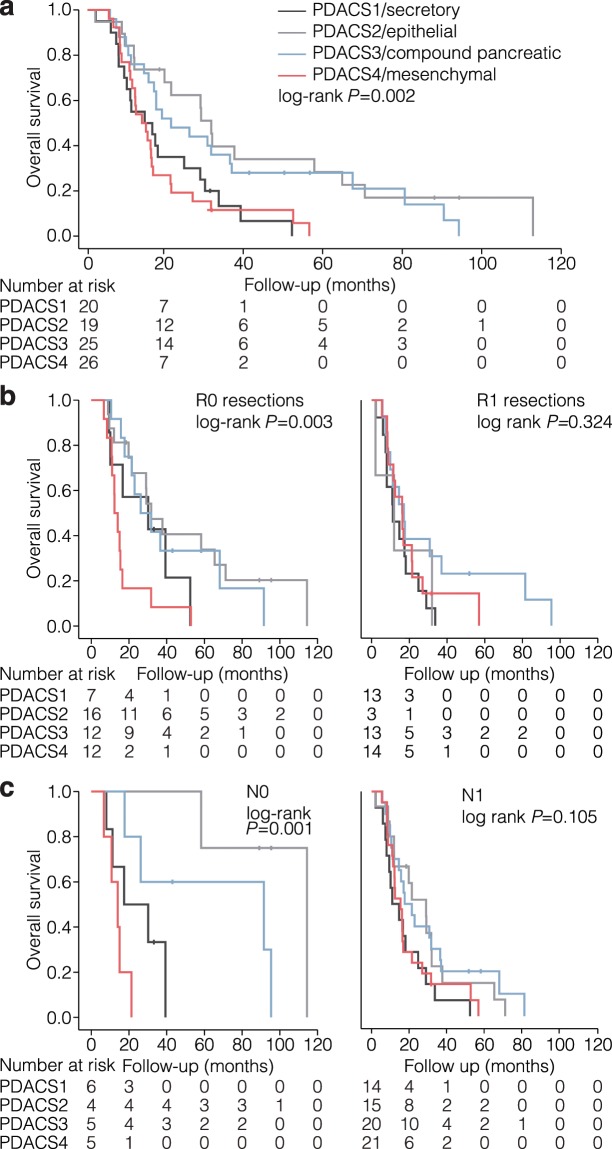
PDACS groups associate with outcome. (**a**) Association of PDACS groups with overall survival was analyzed by Kaplan-Meier and log rank testing. Number at risk per subtype are indicated below. (**b**) As for panel a, separated by radicality of resection. (**c**) As for panel a, separated by lymph node metastasis status.

Survival analysis with a 2-year follow-up revealed that the secretory and mesenchymal subtypes were associated with much poorer outcomes (median overall survival (OS) 14.7 and 14.0 months, respectively; Fig. 3a), compared with the epithelial and compound pancreatic subtypes (median OS 31.8 and 21.5 months). Pairwise comparison of OS between the subtypes showed that the epithelial and the compound pancreatic subtypes differed significantly from the mesenchymal subtype (Supplementary Table 2). Kaplan-Meier survival analysis revealed that radicality of resection (*P* = 0.007), lymph node status (*P* = 0.003) and degree of differentiation (poor vs well *P* = 0.011; poor vs moderate *P* = 0.002) also associated with outcome (Supplementary Fig. 3). We performed multivariate Cox proportional hazard regression analysis to test the statistical significance of the association of PDACS with OS, adjusting for potential confounders including sex, age at diagnosis, radicality of resection, lymph node metastasis and differentiation grade (Supplementary Table 3). The association of the secretory and mesenchymal subtypes with poor outcome remained significant (*P* = 0.018 and P = 0.036, respectively).

Of note, the correlation of PDAC subtypes with outcome was most apparent in patients with confounding variables associated with favorable outcomes: In the small group of patients with complete radicality of resection (R0), the mesenchymal subtype associated more strongly with poor outcome than any other subtype (median OS 17.2 months, *P* = 0.004 pairwise comparison with secretory subtype; Fig. 3b). In patients with no evidence for lymph node metastasis (N0), the mesenchymal subtype was also strongly correlated with poor outcome (Fig. 3c; median OS 14.0 months, *P* = 0.010 pairwise comparison with secretory subtype).

To assess whether our PDACS classifier could identify subgroups with poor survival in other cohorts, we merged published expression datasets and classified samples as mesenchymal (PDACS4) or non-mesenchymal (PDACS1–3). Survival analysis showed that the mesenchymal subtype was also associated with poor outcome in this merged validation cohort (Supplementary Fig. 4a,b)^20,32^.

### Existing classification systems are interconnected

**Figure 4:**
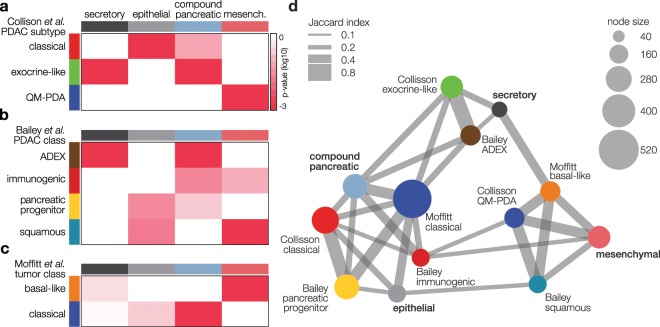
Existing classification systems are interconnected. (**a–c**) Expression data from the AMC cohort were pooled with those from previously established pancreatic cancer classifications^20,21,25^, and subtyped using the PDACS classifier (columns) and published methods (rows). Heatmaps indicate statistical significance of classification agreement. For the Moffit *et al*. classification, tumor subtypes were included. n = 714 samples in total. Colors correspond to published labeling. QM-PDA, quasi-mesenchymal pancreatic ductal adenocarcinoma; ADEX, aberrantly differentiated endocrine exocrine. (**d**) Network analysis of the interconnectivity between classification systems. Each node corresponds to a subtype and size indicates number of samples classified as such. Colors correspond to the published labeling as for panels a–c. Line (edge) thickness indicates the strength of association quantified by Jaccard similarity coefficient.

To further validate our classifier, we compared it to existing classifiers by applying it to samples pooled from our samples and previous studies (Supplementary Table 4). We summarized the statistical significance of association between subtypes in heatmaps (Fig. 4a–c). This approach revealed key correlations among secretory, epithelial, and mesenchymal PDACS with published subtypes. For example, the secretory subtype significantly correlated with subtypes identified in two other studies that share the exo- and endocrine features of the pancreas. The epithelial PDACS also strongly correlated with subtypes from two other studies that represented classical, epithelial tumors (Figs. 4a and 2b, respectively). We also tested all four pancreatic cancer classifications on the pooled samples in a combined network analysis. This analysis showed that our PDAC subtypes and those previously published are highly interconnected and revealed the existence of three major clusters (Fig. 4d). Given its strong association with poor outcome and consistent interconnection with similar identified subtypes, we focused on the mesenchymal subtype and its comparison to the other (non-mesenchymal) subtypes for further experimentation and analysis.

### Classification of models for PDAC reveals that mesenchymal features are tumor-cell intrinsic

To study the molecular mechanisms that underlie the poor outcome of the mesenchymal tumor subtype, and discover potential vulnerabilities, we attempted to use the PDACS classifier to identify experimental models that mimic this specific subtype. We found that the patient PDACS classifier did not perform well on non-patient samples, as PDX samples and cell lines were not consistently assigned to subtypes, and that the classifier required modification for use on such models. We had expected this given the extrinsic factors that affect gene expression, especially in PDAC where extrinsic stroma factors have a large impact on tumor biology and substantially differ in these models from the host.

**Figure 5:**
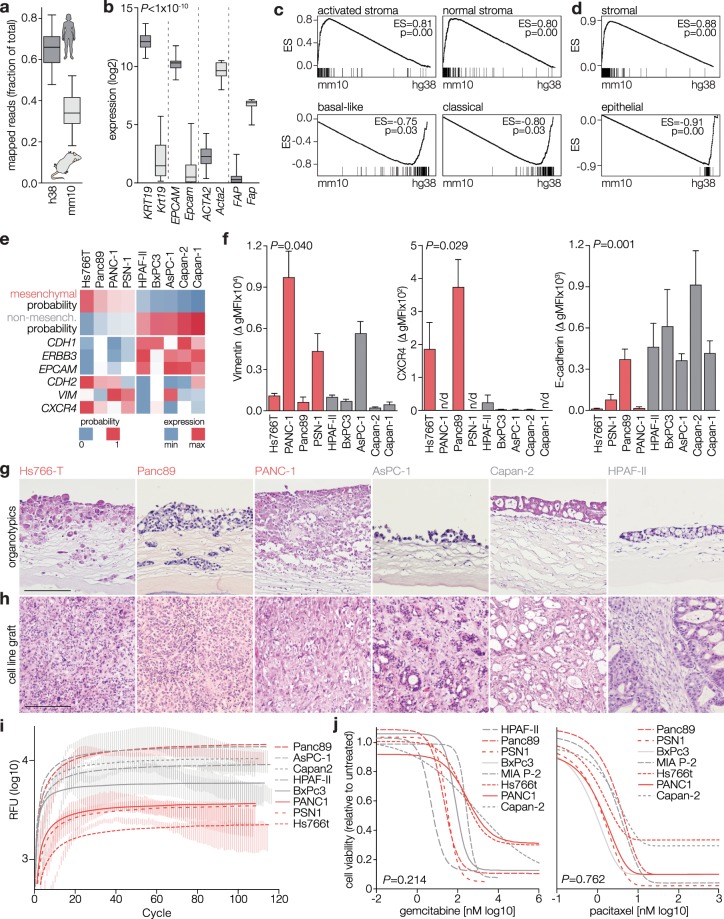
Classified models for PDAC reveal subtype-intrinsic tumor biology. (**a**) Fraction of RNA-Seq reads from PDXs unambiguously mapped to the human (hg38, dark grey) and mouse genome (mm10, light grey). Data are shown from 14 PDXs. (**b**) Expression of epithelial and stromal markers in the human and mouse reads indicated by gene nomenclature (all uppercase, human). All comparisons were significant at the indicated *P*-value determined by t-test. Sample size as for a. (**c**) GSEA comparing mouse- and human-mapped PDX gene expression. Mouse reads are included as samples grouped left in the phenotype ranking. Human reads are samples to the right. Gene sets used were the Moffitt *et al*. activated and normal stroma, and basal-like and classical tumor factor genes. ES, enrichment score. (**d**) As for panel c, using the ESTIMATE stromal and epithelial gene sets. (**e**) Heatmap representation of probability scores for commonly used cell lines. See Supplementary Fig. 5e for a complete overview, of note is that the human reads from the analysis shown in panels a-b were used to generate this epithelial cell-tailored classifier. Probabilities for the non-selected subtype (i.e. the probability for non-mesenchymal subtype of a mesenchymal cell line) was calculated by subtracting the highest ranking score from 1 to yield the inverse. (**f**) Flow cytometry for indicated markers was performed on indicated cell lines. T-test on pooled data from the mesenchymal versus non-mesenchymal cells yielded the *P*-values shown in the panels. (**g**) Mesenchymal (red labels) and non-mesenchymal (grey labels) cell lines grown in organotypic cocultures with stellate cells for 3 weeks. Histology was then assessed. Equal magnifications used for all images; scale bar, 200 μm. (**h**) Indicated cell lines were subcutaneously grafted in NSG mice. Tumors were harvested for histological assessment. (**i**) Transwell migration assay with the indicated cell lines showing baseline chemokinesis (i.e. movement without chemoattractant gradient). n = 3. (**j**) PDACS classified cell lines were treated with indicated concentrations of gemcitabine or paclitaxel (Selleck, Houston, TX). Viability was measured by MTT. Curves were fitted with non-linear regression, dose response curves. *P*-values (by ANOVA) shown for pooled mesenchymal cell lines (n = 12 independent measurements on indicated lines) versus pooled non-mesenchymal cell lines (n = 15). For paclitaxel treated cells, mesenchymal cell lines (n = 12) and non-mesenchymal cell lines (n = 6).

To generate a PDX classifier, we assembled a tumor cell-specific and a stroma-specific classifier using 14 PDXs derived from the patient cohort: RNA-Seq reads from these PDXs were mapped to the human and mouse genome to allocate the expression of genes to the tumor or stromal compartment (Supplementary Fig. 5a). The fraction of reads mapped to either genome is shown in Fig. 5a and Supplementary Fig. 5b. Correlations of gene expression in unmatched tumor-PDX samples as well as matched donor-PDX pairs are shown in Supplementary Fig. 5c,d). As expected, genes associated with stroma were almost exclusively found in the mouse reads (*Fap*, *Acta2;* Fig. 5b), and tumor marker expression was found in reads mapped to the human genome. GSEA with the Moffitt *et al*. and ESTIMATE stromal gene sets^30^ revealed strong enrichment in expression assigned to the mouse genome (Fig. 5c,d), providing further support for the validity of our species-specific transcript analysis.

We then used the human reads from the PDXs, in conjunction with the consensus clustered patient data, to train new epithelial classifiers and identify mesenchymal PDXs versus non-mesenchymal (secretory, epithelial, and compound pancreatic grouped; workflow shown in Supplementary Fig. 5a, classifier genes shown in Supplementary Table 5). We generated probability scores for mesenchymal subtype and a ranking of PDXs shown in Supplementary Fig. 5e. A heatmap comparison of PDX classification and donor subtype is shown in Supplementary Fig. 5f. Of note, this revealed a degree of incongruence between patient and PDX classification. We take this to imply high plasticity in pancreatic cancer tissue and cell states that is context dependent, and further underscores the need to apply experimental model-specific classification methods.

For the cell lines, we used a similar strategy and assembled a classifier for previously established cell lines^33^. We classified Hs766T, Panc89, PANC-1, and PSN-1 as mesenchymal, and HPAF-II, BxPC3, AsPC-1, Capan-2, and Capan-1 as non-mesenchymal (probability scores shown as heatmap in Fig. 5e, see also Supplementary Fig. 6 for classification of all cell lines). Mesenchymal classification strongly correlated with the expression of mesenchymal identity markers (Vimentin) and invasive growth (CXCR4) as determined by gene expression (Fig. 5e) and flow cytometry (Fig. 5f). Conversely, non-mesenchymal cells highly expressed E-cadherin, *EpCAM* and *ERBB3/HER3*.

To determine the functional phenotype of mesenchymal classification, we grew classified cell lines in organotypic cocultures with pancreatic stellate cells^34^. Importantly, we observed invasive growth patterns for all mesenchymal cell lines (Fig. 5g). In organotypic cultures with non-mesenchymal cell lines, we observed a relatively well-demarcated and differentiated non-invasive epithelial layer. Moreover, tumors grown *in vivo* from mesenchymal subtype cell lines exhibited poor differentiation compared to non-mesenchymal tumors (Fig. 5h). Using Transwell migration assays, we verified that this invasive growth was not due to enhanced chemotactic capacity (Fig. 5i). Since we showed that invasive growth is a key feature of mesenchymal PDAC cells, our experiments provide important evidence for the functional relevance of our gene expression-based classification. In addition, we did not find differences in the sensitivity to gemcitabine or paclitaxel between mesenchymal and non-mesenchymal cell lines (Fig. 5j), arguing that the invasive growth is responsible for poor outcome, rather than differential sensitivity to commonly used chemotherapeutics against PDAC.

## Discussion

We have described gene expression analysis and unsupervised class discovery on a well annotated single-center PDAC-only RNA-Seq expression dataset. These analyses revealed the existence of four subgroups of PDAC that correlate with distinct clinical manifestations. By characterizing the associated tumor biology *in silico*, as well as in matching patient-derived models and classical cell lines *in vitro* and *in vivo*, we found that a highly invasive growth pattern, intrinsic to the tumor cell compartment, is associated with poor-prognosis PDAC.

Despite the known contributions of the mutational spectrum to tumor heterogeneity, we did not uncover enrichments for specific mutations in the PDAC subgroups. Given the large influence that the stroma exerts on tumor cell behavior, it is likely that tumor cell-extrinsic factors contribute significantly to gene expression-based analyses of PDAC outweighing the contributions of tumor cell-intrinsic mutations^25^. Furthermore, a recent publication describing the epigenetic determinants of PDAC subtypes suggests that a certain degree of plasticity exists between these molecular subgroups^35^. This plasticity was further supported by a limitation of our study: the discordance that we observed between PDXs and their donors at the gene expression level. We hypothesize that this is indeed due to the large stromal impact on tumor cell gene expression. We have previously observed that already within the first passage after grafting, nearly all stromal cells are of mouse origin^36^. The composition of this mouse stroma, and the influence it exerts on the grafted tumor cells, is different from the original human stroma. This affects the accuracy with which the gene expression profiles of tumor cells, grown in different host species, reflect those from patients. This is in line with a recent study showing that specific macrophage populations in the tumor microenvironment drive squamous subtype tumor biology^37^. In addition, it is possible that the subcutaneous site of PDX growth hampers comparison to the original site of growth in the human pancreas. Nonetheless, the use of species-specific transcript analysis of PDXs has allowed successful classification of experimental models of PDAC and revealed strong correlations of the mesenchymal subtype with invasive potential *in vitro* and high-grade tumor growth *in vivo*.

Additionally, the question rises why the secretory subtype is associated with poor outcome in our full cohort (including R1 and N1 patients). Based on previously published subtyping studies, the mesenchymal subtype would be expected to unequivocally predict poor outcome, but in our cohort this was only apparent when considering the R0 and N0 patients^20,21,25^. Network analysis revealed the secretory subtype to correlate with the previously described ADEX/exocrine-like subtypes and these are not known to associate with relatively poor prognosis. A tentative explanation is that poor outcome in our mesenchymal subtype becomes most apparent when clinical confounders for poor outcome are considered in depth, and that in these selected patients, the tumor cell-intrinsic properties of the ADEX/exocrine-like subtypes do not contribute to poor outcome. Tumors that grow in the tail of the pancreas have been suggested to be of mesenchymal/squamous subtype relatively often^38^. However, in the 90 samples available in our cohort, only two originated from the pancreas tail precluding meaningful analysis on clinical variables and associations to our molecular subtypes.

Our compound pancreatic subtype appeared to identify similar samples as did the previously published classical and ADEX/exocrine-like subtypes. However, the biology of this subtype as revealed by GSEA for KEGG and GO signatures was decidedly shared with the *mesenchymal* subtype (Fig. 2 and Supplementary Fig. 2), and we propose that the compound subtype does not result from inappropriate classification, but rather from intratumor heterogeneity, where the presence of more than one PDACS subtype (one of which mesenchymal) within the tissue analyzed results in a subtype that is characterized by features that differentially impact on its biology and classification.

The parallels between the subtypes identified by our own as well as previously published classifiers, suggest that a unifying transcriptome-based classification for PDAC can be accomplished. Whether this classification should be based solely on the transcriptome of tumor cells, or also include signatures derived from cells within the tumor microenvironment is currently still a matter of debate. Achieving consensus on the number and the identity of gene expression-based subtypes will aid future clinical implementation^39^. Such an effort in PDAC could also be extended to include other periampullary tumors, and could even cover the full width of gastrointestinal cancers^40^. This would greatly simplify the application of a molecular classifier, and with careful design, at a minimal cost for the detection of rare or organ-specific subtypes^41^.

We identified PDAC subtypes defined by distinct tumor biology and clinical manifestations. Our subtypes show high interconnectivity with previously published classifications. Our data indicate that the very limited surgical benefit for patients bearing the most aggressive tumor subtype argues against direct surgical resection of such tumors even if otherwise favourable clinical features are present. This poor prognosis following resection is caused by the infiltrative growth pattern rather than a difference in response to chemotherapy.

## Methods

### Clinical data, tissue collection, and ethical approval

Tumor tissue of patients who underwent a pancreaticoduodenectomy (PD) for a PDAC at the Amsterdam UMC, location Academic Medical Centre Amsterdam (AMC) between 1993 and 2015 was retrospectively collected from the fresh frozen tissue archive of the Department of Pathology (n = 230), and from the prospectively collected cohort of the Laboratory for Experimental Oncology and Radiobiology (BioPAN; n = 115^36^). Retrospective collection was conducted in accordance with ethical guidelines ‘Code for Proper Secondary Use of Human Tissue in The Netherlands’ (Dutch Federation of Medical Scientific Societies), approved by the Academic Medical Center’s institutional review board (Medisch Ethische Toetsingscommissie AMC) under METC_A1 15.0122. For prospectively collected material, informed consent was obtained from all patients in accordance with our hospital’s ethical guidelines (IRB code METC 2018_181). All specimens were snap-frozen in liquid nitrogen and stored at −80 °C. Clinicopathological data were obtained through the departments of Surgery and Pathology and expanded with parameters to include age, sex, type of surgery, chemo(radio)therapy regimen, radicality of surgery, size and differentiation grade of the tumor, and overall survival (Table 1). Total follow-up was over 120 months and median follow-up for living patients was 52 months (range 19–120), and 16 months (range 2–95) for deceased patients. For histopathological revision, selection, and processing of PDAC samples see Supplementary Methods.

### Preparation of libraries and processing for RNA-seq

For RNA isolation, 30 sections of 20 μm were cut, and RNA was isolated using RNABee (Bio-Connect, Huissen, the Netherlands) and the RNeasy Mini kit (Qiagen, Hilden, Germany) according to manufacturer’s instructions. In most samples the RNA Integrity Number was 7 or higher as evaluated by BioAnalyzer (Agilent, Santa Clara, CA), median RIN = 8. Of 19 samples the RIN value was under 7 but this was not apparent from principle component analysis (PCA) of the gene expression profiles. Samples were DNAse-treated. RNA was amplified using the Total Prep RNA Amplification kit (Illumina, San Diego, CA). Poly-A enriched libraries were synthesized using TruSeq RNA Library Prep kit and sequenced in three batches (Illumina HiSeq2500). All sequencing data were quality-controlled using FastQC^42^ and found to be of high quality. RNA-Seq reads were aligned to the human reference genome (GRCh38) using Tophat2 (V2.1.0^43^) with default parameters, retaining only uniquely mapped reads. Gene expression levels were estimated using Cufflinks (V2.2.1), with default parameters and Gencode V19 for gene annotation, masking rRNAs, tRNAs and chromosome M. The resulting gene expression profiles, measured by RPKM (reads per kilobase of transcript per million mapped reads) were log2-transformed. Non-biological batch effects were inspected using PCA, and corrections were made using Combat^44^. Subsequent analyses were done on the batch-corrected dataset.

### Identification of PDAC subtypes

In order to identify PDAC subtypes, genes with average log2 (RPKM) > 1 and a median absolute deviation (MAD) > 0.5 across samples were retained and median-centered. Hierarchical clustering with agglomerative average linkage was used for unsupervised classification. To evaluate the stability of clustering, consensus clustering was employed^45^, with 1000 iterations and 95% subsampling ratio. A significant increase in clustering stability was observed from k = 2–4, but not for k > 4 (Supplementary Fig. 1a–c). Gap statistics^46^ were calculated for k = 1–8, and a peak was found at k = 4, confirming four robust clusters (Supplementary Fig. 1d).

### Generation of the PDACS classifier

To build the PDAC Subtype (PDACS) classifier, we applied two filtering steps to select the most differential and discriminative genes. First, we identified genes significantly differentially expressed (false discovery rate, FDR < 0.01) between each PDAC subtype and the other three using significance analysis of microarrays (SAM; R package ‘siggenes’, V1.44.0^47^). Second, for each subtype we selected top 40 discriminative genes based on AUC (area under receiver operating characteristics, ROC curve, R package ‘ROCR’ V1.0–7;^48^. The resulting 159 genes in total were trained by prediction analysis for microarrays (PAM;^28^) to build a classifier. The classifier was used for classification of samples in the other data sets (Bailey, PACA-AU and TCGA), where we regarded the subtype with the highest posterior probability as being indicative of association with that group. To facilitate classification of gene expression data sets generated from other platforms, we filtered gene expression data by taking the commonly annotated genes between the sets analyzed. For analysis of public patient data sets, cross comparisons between PDAC classification systems, gene set analysis see Supplementary Methods.

### Cross comparisons between PDAC classification systems

The strength of pairwise associations between subtypes of different PDAC classification systems was statistically assessed. We first reproduced the classifier used by each subtyping system based on corresponding signature genes and the discovery data set. Subsequently, we performed cross-classifications, i.e. to use each classifier on the discovery data sets of all reported subtyping systems. For each two classification systems, sample enrichment analysis was performed using hypergeometric tests to compare their corresponding classification results. Benjamini-Hochberg (BH) corrected *P*-values were derived from these tests, indicating the strength of association between the studied two classification systems.

To further systematically elucidate the interrelations between all PDAC classification systems, we employed a network-based meta-analysis approach that was established by us previously^39^. The network encodes on nodes the information of subtype prevalence and on edges their association calculated by Jaccard similarity coefficient, which is defined by the size of the intersection between two sample sets over the size of their union. To quantify the statistical significance of subtype associations, we performed hypergeometric tests for overrepresentation of samples classified to one subtype in another. The resulting *P-*values were adjusted for multiple hypotheses testing using the BH method. Using this approach, we built a network consisting of subtypes defined in all subtyping systems, interconnected by statistically significant (BH-corrected, *P* < 0.001) edges.

### Statistical analysis

Clinicopathological parameters were collected and analyzed in SPSS V24 (IBM, Armonk, NY). Statistical testing on continuous variables (>2 groups) were done by ANOVA, and Pearson Chi-square (χ^2^) tests for categorical variables. Hypergeometric tests were used to quantify the statistical significance of association between subtypes of different classification systems. To analyze OS, we used the Kaplan-Meier (KM) method, with log-rank tests for calculation of p-values. Multivariate Cox proportional hazards regression analysis was used to test the statistical significance of PDACS subtypes with survival, adjusting for potential confounders (STATA, StataCorp, College Station, TX). Statistical testing on *in vitro* data was performed using Graphpad Prism 7. Comparing mesenchymal versus non-mesenchymal groups with continuous variables were either tested by t-test for Gaussian distributed values or by Mann-Whitney U test for non-Gaussian distributed values.

### PDX sequence analysis

PDX samples (n = 14, from 13 patients) were processed and sequenced as the patient biopsies. Sequence reads from PDX samples were mapped to the mouse (mm10) and human genomes (hg38) and assigned to either one using XenofilteR^49^. Xenograft-donor matching was confirmed by short tandem repeat (STR) profiling (Promega, Madison, WI) of the donor patients’ gDNA (isolated from blood) and gDNA isolated from the PDX. Gene set enrichment analysis on merged mouse and human sets (Fig. 4c,d) was performed using the software available through the Broad institute website, using 1000 permutations on the phenotype^50^. For generation of patient-derived xenografts and cell lines see Supplementary Methods.

### Donor-PDX expression comparison

To be able to compare expression profiles from PDX models (which are a mix of expression originating from the transplanted human tumor and murine stromal infiltration from the host) to donor tumors we combined the expression originating from both compartments. First, using the ‘biomaRt’ R package we mapped human genes to their best mouse homologs. Secondly, using this mapping, we summed reads from mouse and human origin to obtain a combined expression value per gene. Finally, these expression values were normalized to reads per million and log2 transformed. For the calculation of the donor-PDX correlation coefficients, genes were selected that showed similar expression behavior in patient tumors and PDX models. For this we used a correlation of correlations approach, only selecting genes with a coefficient above 0.25 (n = 5039)^39^. This selection was further refined by only including sufficiently variable genes with a standard deviation above 1.7 in both the donor and the PDX dataset (n = 113).

### Epithelial and stromal PDX classifiers

Linnekamp *et al*. showed that they were able to subtype PDX models derived from colorectal tumors using an epithelial classifier^51^. Using a similar approach we built an epithelial and stromal PDACS classifier. To identify tumor-specific genes that were expressed in the epithelial compartment, but not or much less in the stromal compartment (and *vice versa* for stroma-specific genes), we used the RNA-Seq profiles of our PDAC PDX models (using the human-mouse homology mapping described in the previous paragraph). We calculated the minimum and maximum expression in the epithelial compartment (min.ep and max.ep) and stromal compartment (min.str and max.str) across all samples for each gene and selected genes based on the following criteria: epithelial genes = (min.ep > 1.5 & max.str < 1.5 & min.ep - max.str > 1.25) or min.ep - max.str > 5; stromal genes = (min.str > 1.5 & max.ep < 1.5 & min.str - max.ep > 1.25) or min.ep - max.str > 8.

These two sets of genes were subsequently used to train an epithelial and a stromal PDACS classifier, able to distinguish mesenchymal samples (PDACS4) from the other subtypes (PDACS1–3) (Supplementary Table 5). Input for this procedure was the AMC patient dataset, excluding PDX-donor patients to avoid bias. Before classifier construction the expression was normalized using the method described in Linnekamp *et al*. to counteract the tumor and stromal dilution effects, after which a support vector machine (SVM) classifier was constructed with the e1071 R package using a linear kernel. The PDX models were then classified using the human and mouse expression components for the epithelial and stromal classifier respectively. Of note, a 4-tier classification of PDAC cell lines was not successful. For cell line classification, reagents, *in vitro* assays, flow cytometry, organotypic cocultures and migration assays, see Supplementary Methods.

## Supplementary information

Supplementary Information.

Supplementary Table 1.

Supplementary Table 4.

Supplementary Table 5.

Supplementary Table 6.

## Data Availability

RNA-Seq data have been deposited at EMBL-EBI ArrayExpress (E-MTAB-6830).
