# Supplementary material for: Unsupervised class discovery in pancreatic ductal adenocarcinoma reveals cell-intrinsic mesenchymal features and high concordance between existing classification systems
Source: Sci Rep. 2020 Jan 15;10:337. doi: 10.1038/s41598-019-56826-9 (PMC6962149; doi:10.1038/s41598-019-56826-9)
Supplement: Supplementary file 1 — Supplementary Information. [file 41598_2019_56826_MOESM1_ESM.pdf]

Supplementary information for:

Unsupervised class discovery in pancreatic ductal adenocarcinoma reveals cell-intrinsic mesenchymal features and high concordance between existing classification systems

Authors:

Frederike Dijk, Veronique L. Veenstra, Eline C. Soer, Mark P.G. Dings, Lan Zhao, Johannes B. Halfwerk, Gerrit K. Hooijer, Helene Damhofer, Marco Marzano, Anne Steins, Cynthia Waasdorp, Michal Heger, Olivier R. Busch, Marc G. Besselink, Johanna A. Tol, Lieke Welling, Lennart B. van Rijssen, Sjors Klompmaker, Hanneke W. Wilmink, Hanneke W. van Laarhoven, Jan Paul Medema, Louis Vermeulen, Sander van Hooff, Jan Koster, Joanne Verheij, Marc J. van de Vijver, Xin Wang & Maarten F. Bijlsma

Supplementary methods

Histopathological revision, selection, and processing of PDAC samples

Frozen sections of 5 μm were stained with haematoxylin and eosin (HE) and examined by two independent pathologists (J.V. and M.J.V.) to confirm diagnosis and assess tumour cell percentage [1]. Twenty-six samples were excluded because of non-PDAC diagnosis. PDAC samples that contained less than 30% tumour cells after macrodissection were excluded from further processing, with the exception of cases from which patient-derived xenografts were established. From 345 available samples, 90 cases were appropriate for RNA isolation (62 retrospectively, and 28 prospectively collected samples).

In vitro assays

Hs766T (ATCC), PSN-1 (kind gift from Prof. Peblani, University of Padova, Italy), PANC-1 (ATCC) were cultured in DMEM. Panc89 (kind gift from Prof. Knippschild, University hospital Ulm, Germany), BxPC3 (ATCC), AsPC-1 (ATCC), were cultured in RPMI. Capan-1 (ATCC), Capan-2 (ATCC), HPAF-II (ATCC), and all primary cell lines were cultured in IMDM. PS-1 stellate cells were cultured in DMEM/F12 and intermittent puromycin selection [2]. All media were supplemented with 8% FCS, L-glutamine (2 mM), penicillin (100 units/mL), and streptomycin (500 µg/mL) and all cells were cultured according to routine procedures. All cell lines were authenticated by short tandem repeat (STR) profiling (Promega, Madison, WI) and comparison to the Expasy Cellosaurus database, and tested for mycoplasma by PCR monthly. For drug response assays measured by MTT (Fig. 5j), cells were seeded at 2000 cells/well in 96-well plates. After 3h, cells were treated for an additional 4 days and MTT was added for 3h prior to measuring.

Organotypic cocultures

Organotypic cocultures were assembled as previously described [2]. Tumour cells and pancreatic stellate cells (PS-1) were plated in a 1:2 ratio on Matrigel/collagen cushions solidified on nylon mesh placed on metal grids. Cells were cultured at the air-liquid interphase. Medium was replaced twice a week. After culturing for 3 weeks, cultures were processed for immunohistochemistry (IHC) by fixation with 4% paraformaldehyde and processed according to standard procedures for paraffin embedding.

Migration assays

Transwell migration assays were performed using FluoroBlok inserts with 8.0μm pore filters (Corning, Corning, NY) as described in ref [3]. In brief, cells were incubated with 10μM Cell Tracker Green for 1h (Invitrogen, Carlsbad, CA) in serum-free medium, and equilibrated in serum-free medium for 1h. 600μl medium was placed in the lower compartment of the plate. Cells were collected, suspended in serum-free medium at 5.0×10^4 cells in 100μl, and seeded into the upper compartment of the Transwell insert. Fluorescent signal from the bottom surface of the insert was measured every 2 minutes for 3h using a

cytofluormeter (BioTek Instruments, Winooski, VT). Temperature was kept at 37°C. Migration was controlled for background.

Flow cytometry

Prior to staining for flow cytometry, cells were harvested with trypsin and washed in FACS buffer (PBS with 1% FCS). Cells were analysed with 1 μg/ml PI on a FACSCanto II (BD, Franklin Lajes, NJ). Data were analysed using FlowJo v10 (FlowJo, Ashtree, OR).

Generation of patient-derived xenografts and cell lines

Collection of patient material for grafting was approved by the institute's pertinent ethical committee (AMC BTC 2014_181), and performed according to the Helsinki Convention guidelines. Written informed consent was obtained for all inclusions. Grafting of immune deficient *NOD-scid IL2Rgamma*^{null} (NSG) mice with patient material was performed according to procedures approved by the animal experiment ethical committee (DTB102348/LEX268). Mice of both sexes, ranging in age from 2 to 9 months, were used. All surgical procedures were performed under isoflurane anaesthesia. Interventions were done during daylight hours. Mice were housed in filtertop cages with enrichment (cardboard rodent homes). Mice were not fasted, and fed a*d libitum*. See also reference [4].

Analysis of public patient data sets

In addition to our in-house RNA-Seq data set, we analysed three other independent gene expression data sets for this study (Supplementary Table 4). In short, the Bailey set (GSE36924) contains gene expression data for 91 pancreatic cancer samples based on Illumina HumanHT-12 V4.0 expression bead chips, of which 70 PDAC samples were used for this study. The normalized gene expression profiles were downloaded [5], and ENSEMBL IDs were converted to official gene symbols using the 'biomaRt' package [6]. The PACA-AU set comprises in total 461 samples, of which 269 samples with normalized gene expression data were downloaded and analysed in this study. TCGA PAAD gene expression profiles (n=184) were downloaded from the Firehose Broad GDAC portal (http://gdac.broadinstitute.org/ accessed on Mar 11, 2018), of which 65 samples of high tumour purity (>30%) were analysed in this study [7]. Scaled estimates in gene-level RSEM results from level 3 RNA-Seq data were converted to transcripts per million (TPM), followed by log2-transformation for further analysis. Entrez IDs were converted to official gene symbols using Bioconductor package 'org.Hs.eg.db'.

Gene Set Analysis

Gene Set analysis was performed by the GeneSetMap (GSM) function in R2 (r2.amc.nl). In short, GSM takes a gene set database and a dataset (e.g. cohort) and calculates a single gene set score for every sample for every contained gene set. These scores are average Z-score values across all of the genes

within a gene set. The gene set score is then used to summarize the different gene sets versus samples in a heatmap representation. The gene set scores can also be used to summarize groups of samples on the basis of an annotation feature (grouping variable such as subtype) to produce summary representations. For the GSEA shown in Figure 5, the KEGG oxidative phosphorylation gene set was modified to exclude V-type ATPases and LHPP.

Cell line classification

Raw cell line expression data were downloaded from four public datasets. These included Maupin et al. (GSE21654)[8], the AstraZeneca collection (GSE57083), the Broad Institute's Cancer Cell Line Encyclopedia (CCLE, GSE36133)[9] and the Sanger Cell Line Project (GSE68950). The datasets were normalised and summarized using robust multiarray analysis (rma). Batch effects were removed from GSE68950 using Combat [10]. To be able to faithfully classify cell line models we constructed a cell line specific classifier. First, we used the selection of epithelial tumour genes described above. To ensure sufficient expression in the cell line models, we subsequently selected only genes with a mean log2 expression > 4 in both the Maupin and the AstraZeneca dataset (n=98). Secondly, these genes were ranked using the AMC patient dataset, which was normalized using the method described in Linnekamp et al. to counteract the stromal dilution effect. The ranking was done in a 5-fold cross-validation where 80% of samples were randomly selected and used to identify the top 25 genes most differentially expressed in PDACS4 patients versus PDACS1/2/3 patients. Differential expression was determined using the limma R package [11] and ranking was done on Benjamini-Hochberg corrected P values. After 1000 iterations we retained genes (n=14) that were selected in at least 900 iterations for final SVM classifier construction using the full AMC dataset as input. This classifier was used to stratify the cell lines from the four cell line datasets.

Supplementary Figures, legends, and Tables

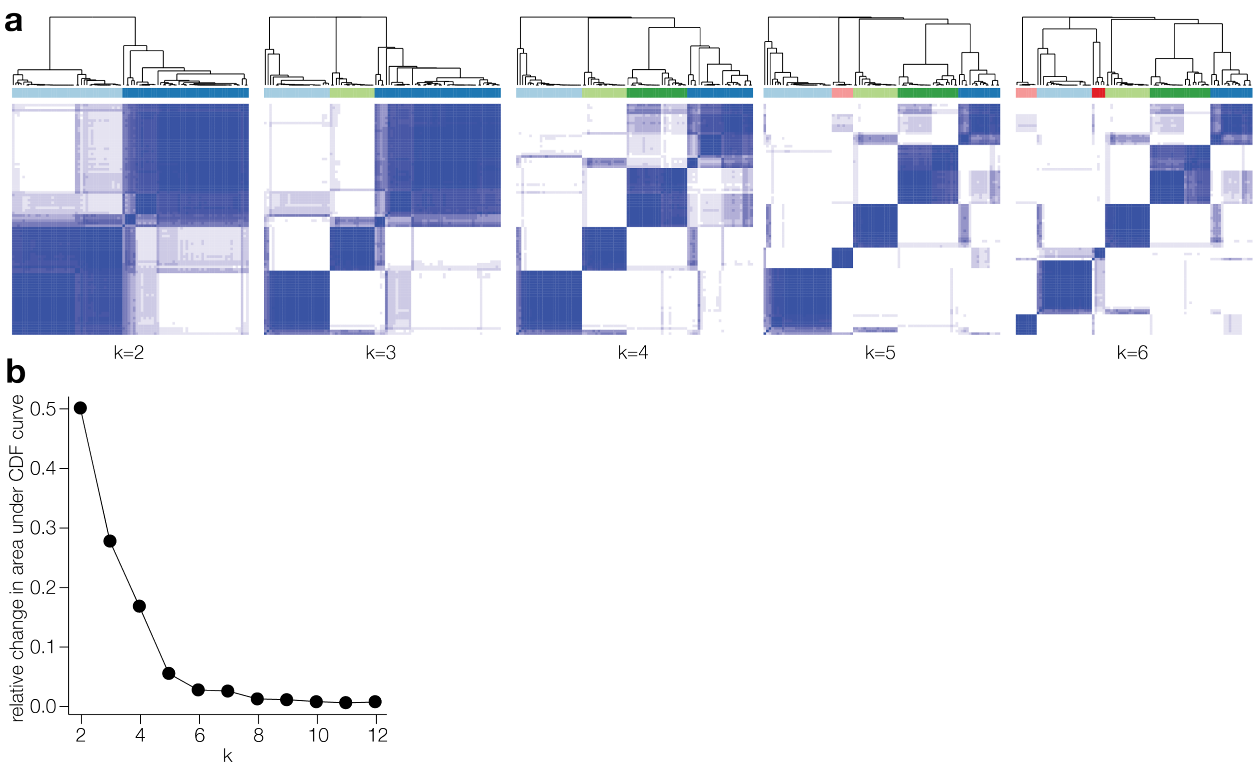

Supplementary Figure 1 l Consensus clustering identifies four robust subgroups of PDAC.

a Consensus matrix heatmaps are shown for indicated cluster numbers (k=2–6).

b Change in area under Cumulative distribution function (CDF) shown in Figure 1.

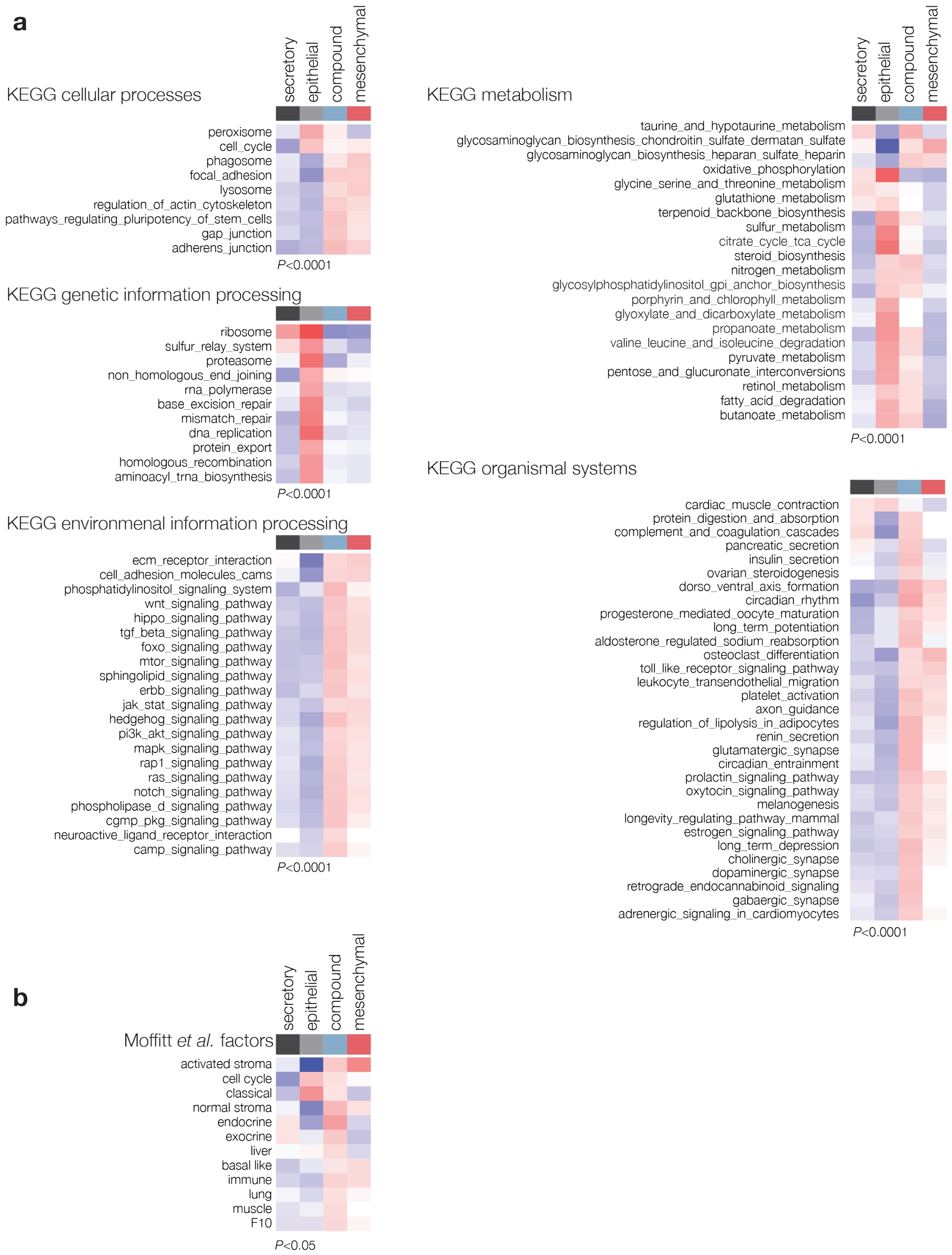
a
KEGG cellular processes
secretory
epithelial
compound
mesenchymal
peroxisome
cell_cycle
phagosome
focal_adhesion
lysosome
regulation_of_actin_cytoskeleton
pathways_regulating_pluripotency_of_stem_cells
gap_junction
adherens_junction
P<0.0001
KEGG genetic information processing
ribosome
sulfur_relay_system
proteasome
non_homologous_end_joining
rna_polymerase
base_excision_repair
mismatch_repair
dna_replication
protein_export
homologous_recombination
aminoacyl_trna_biosynthesis
P<0.0001
KEGG environmenal information processing
ecm_receptor_interaction
cell_adhesion_molecules_cams
phosphatidylinositol_signaling_system
wnt_signaling_pathway
hippo_signaling_pathway
tgf_beta_signaling_pathway
foxo_signaling_pathway
mtor_signaling_pathway
sphingolipid_signaling_pathway
erbb_signaling_pathway
jak_stat_signaling_pathway
hedgehog_signaling_pathway
pi3k_akt_signaling_pathway
mapk_signaling_pathway
rap1_signaling_pathway
ras_signaling_pathway
notch_signaling_pathway
phospholipase_d_signaling_pathway
cgmp_pkg_signaling_pathway
neuroactive_ligand_receptor_interaction
camp_signaling_pathway
P<0.0001
KEGG metabolism
secretory
epithelial
compound
mesenchymal
taurine_and_hypotaurine_metabolism
glycosaminoglycan_biosynthesis_chondroitin_sulfate_dermatan_sulfate
glycosaminoglycan_biosynthesis_heparan_sulfate_heparin
oxidative_phosphorylation
glycine_serine_and_threonine_metabolism
glutathione_metabolism
terpenoid_backbone_biosynthesis
sulfur_metabolism
citrate_cycle_tca_cycle
steroid_biosynthesis
nitrogen_metabolism
glycosylphosphatidylinositol_gpi_anchor_biosynthesis
porphyrin_and_chlorophyll_metabolism
glyoxylate_and_dicarboxylate_metabolism
propanoate_metabolism
valine_leucine_and_isoleucine_degradation
pyruvate_metabolism
pentose_and_glucuronate_interconversions
retinol_metabolism
fatty_acid_degradation
butanoate_metabolism
P<0.0001
KEGG organismal systems
cardiac_muscle_contraction
protein_digestion_and_absorption
complement_and_coagulation_cascades
pancreatic_secretion
insulin_secretion
ovarian_steroidogenesis
dorso_ventral_axis_formation
circadian_rhythm
progesterone_mediated_oocyte_maturation
long_term_potentiation
aldosterone_regulated_sodium_reabsorption
osteoclast_differentiation
toll_like_receptor_signaling_pathway
leukocyte_transendothelial_migration
platelet_activation
axon_guidance
regulation_of_lipolysis_in_adipocytes
renin_secretion
glutamatergic_synapse
circadian_entrainment
prolactin_signaling_pathway
oxytocin_signaling_pathway
melanogenesis
longevity_regulating_pathway_mammal
estrogen_signaling_pathway
long_term_depression
cholinergic_synapse
dopaminergic_synapse
retrograde_endocannabinoid_signaling
gabaergic_synapse
adrenergic_signaling_in_cardiomyocytes
P<0.0001
b
secretory
epithelial
compound
mesenchymal
Moffitt et al. factors
activated stroma
cell cycle
classical
normal stroma
endocrine
exocrine
liver
basal like
immune
lung
muscle
F10
P<0.05

Supplementary Figure 2 | PDACS groups associate with distinct biological programs.

a Enrichment for indicated KEGG gene sets across subtypes are shown. Threshold significance level is indicated below heatmap. Heatmap indicates gene set z-score. Analysis was performed using the gene set maps (GSM) function in R2 (r2.amc.nl).

b Enrichment for the factors identified by Moffitt *et al.* are shown per subtype, as for panel a.

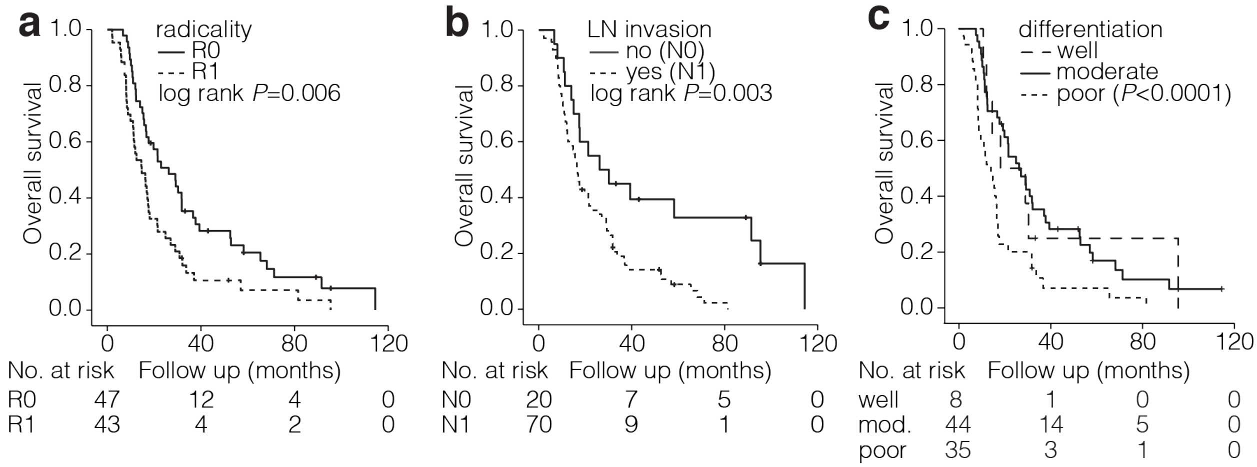

Supplementary Figure 3 l Survival analysis.

a Association of radicality of resection (R0 or R1) with overall survival, by Kaplan-Meier analysis.

b As for panel a, for lymph node metastasis status (N0 vs N1).

c Association of tumour differentiation grade (well, moderate, poor) with overall survival.

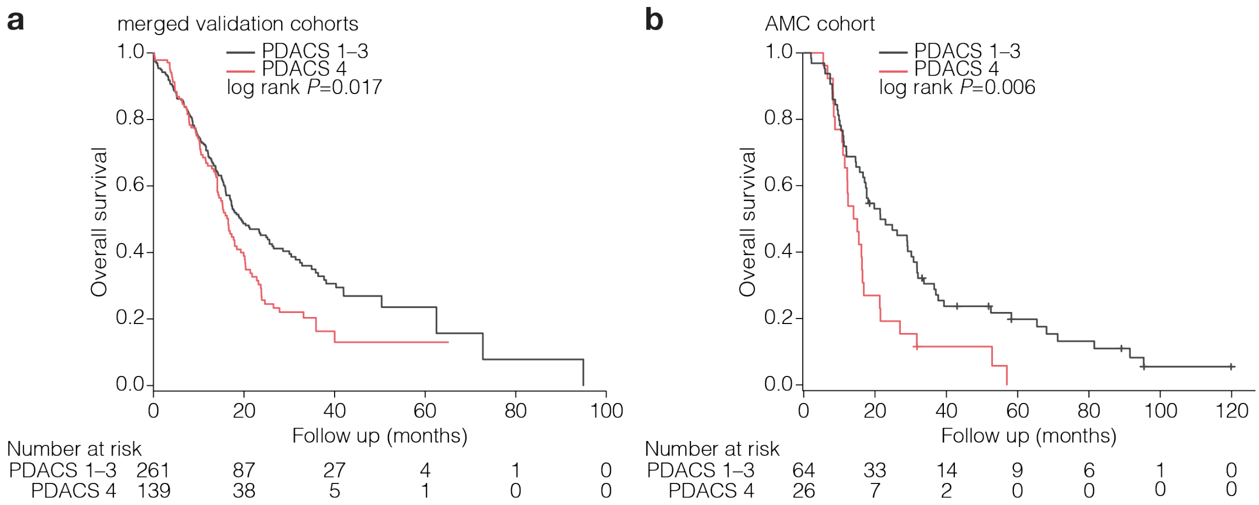

Supplementary Figure 4 l PDAC subtypes associate with survival in validation cohorts.

a Published expression datasets were merged (refs; [5,7] and ICGC), and samples were classified as mesenchymal (PDACS4) or non-mesenchymal (PDACS1-3). Survival analysis was performed using Kaplan-Meier analysis and log-rank test.

b As for panel b, on the AMC cohort.

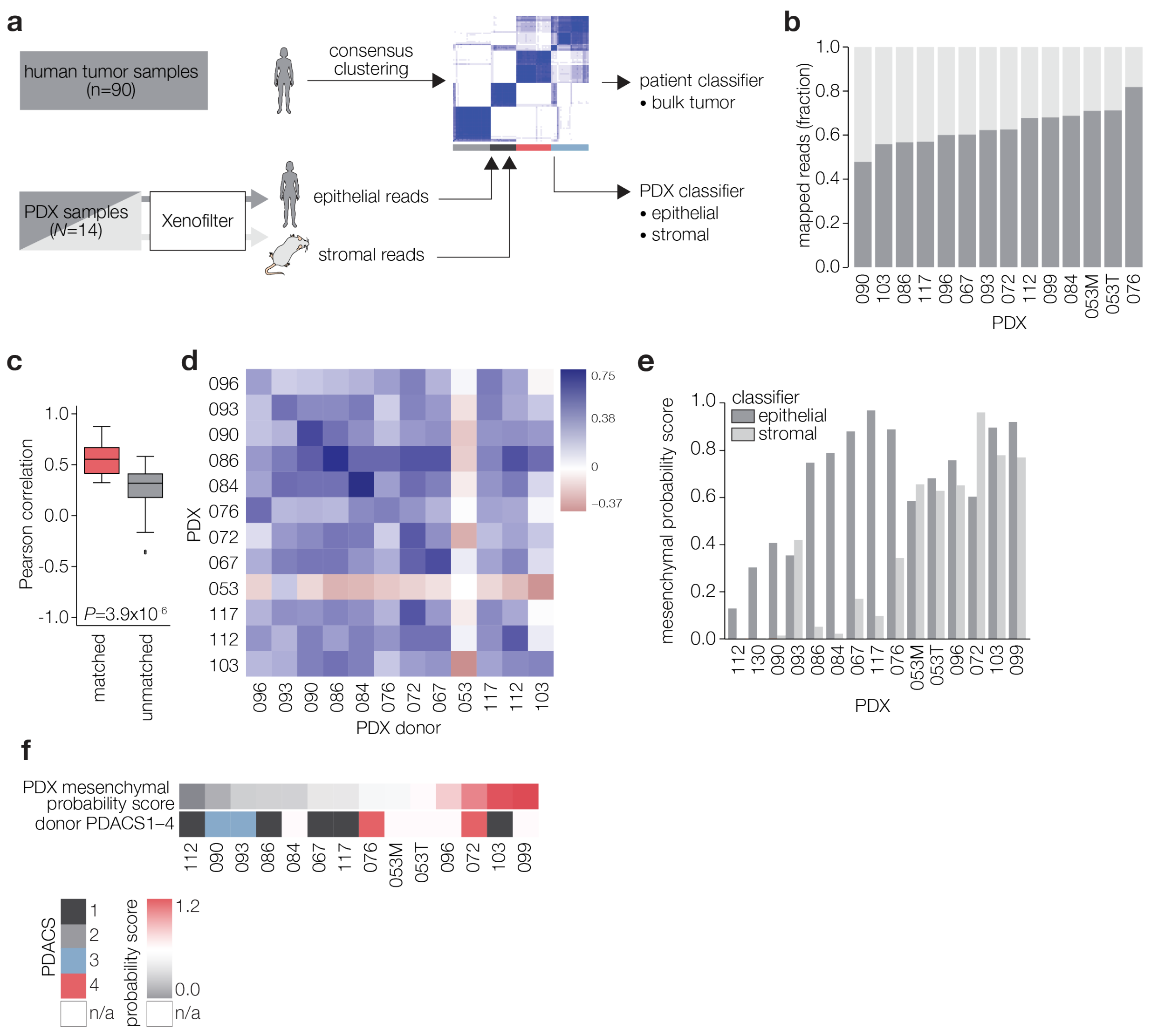

Supplementary Figure 5 | Subtype classified patient-derived xenografts and cell lines.

a Workflow from samples to classifier. 90 human tumour samples and 14 xenograft (PDX) samples were analyzed by RNA-Seq. PDX reads were mapped to human or mouse genome using Xenofilter [12]. Human (epithelial) and mouse (stromal) reads were used in conjunction with the classified patient samples and SVM to assemble compartment specific classifiers. A similar approach was taken using publically available gene expression datasets to classify cell lines.

b Fraction of reads of each PDX mapped to the human (dark grey) or mouse (light grey) genome by Xenofilter.

c Pearson correlation of gene expression between donor-PDX pairs (red), and between all samples (grey). *P*-value shown was obtained by Mann-Whitney test.

d Heatmap representation of gene expression between individual PDXs and their respective donors. Gradient indicates Pearson correlation.

e Probabilities for mesenchymal classification of the PDX models using the epithelial classifier (dark grey bars) and stromal classifier (light grey bars). PDXs ranked by summed probabilities.

f PDX and donor classification. Shown is heatmap representation of sum of probability scores from epithelial- and stromal probability scores as shown in panel e for PDXs. Second row shows PDX donor classification using unmodified patient classifier as show in Fig. 1a.

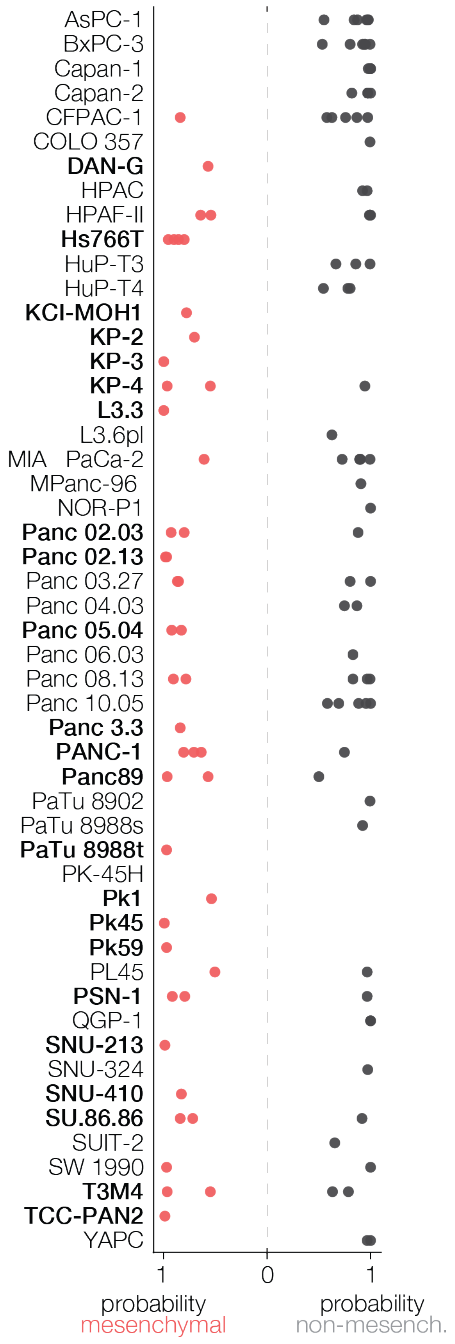

Supplementary Figure 6 | Summary of classification of PDAC cell lines.

Expression data from the GSE57083, GSE36166 [9], E-MTAB-783 [13], and GSE21654 [8] datasets was used. The epithelial PDACS classifier was used. Each dot indicates a classified sample from a dataset, and the relative position on the x-axis indicates probability of classification as mesenchymal (red) or non-mesenchymal (grey).

SUPPLEMENTARY TABLES

Supplementary Tables 1,4, 5, and 6 are provided as Excel files.

Supplementary Table 2 | Pairwise comparison of overall survival per subtype.

	PDACS1 secretory *P*-value	PDACS2 epithelial *P*-value	PDACS3 compound *P*-value	PDACS4 mesenchymal *P*-value
PDACS1		0.011	0.015	0.918
PDACS2	0.011		0.551	0.002
PDACS3	0.015	0.551		0.004
PDACS4	0.918	0.002	0.004	

Supplementary Table 3 | Multivariate analysis of prognostic variables.

		HR	95% CI		*P*-value
subtype	epithelial	1.000			
	secretory	2.707	1.182	6.198	**0.018**
	compound pancreatic	1.116	0.551	2.259	0.76
	mesenchymal	2.235	1.054	4.743	**0.036**
sex	male	1.000	0.646	1.761	0.801
	female	1.067	0.981	1.034	0.607
age at diagnosis		1.007	0.981	1.034	0.607
radicality	R0	1.000			
	R1	1.210	0.729	2.009	0.461
lymph node metastasis	N0	1.000			
	N1	2.036	0.993	4.175	0.052
differentiation grade	well	1.000			
	moderate	1.074	0.430	2.683	0.879
	poor	1.897	0.753	4.780	0.175
	unknown	1.500	0.269	8.376	0.644

SUPPLEMENTARY REFERENCES

1 Soer, E. *et al.* Dilemmas for the pathologist in the oncologic assessment of pancreatoduodenectomy specimens : An overview of different grossing approaches and the relevance of the histopathological characteristics in the oncologic assessment of pancreatoduodenectomy specimens. *Virchows Arch* **472**, 533-543, doi:10.1007/s00428-018-2321-5 (2018).

2 Kadaba, R. *et al.* Imbalance of desmoplastic stromal cell numbers drives aggressive cancer processes. *J Pathol* **230**, 107-117 (2013).

3 Bijlsma, M. F., Damhofer, H. & Roelink, H. Hedgehog-stimulated chemotaxis is mediated by smoothened located outside the primary cilium. *Sci Signal* **5**, ra60 (2012).

4 Damhofer, H. *et al.* Establishment of patient-derived xenograft models and cell lines for malignancies of the upper gastrointestinal tract. *J Transl Med* **13**, 115 (2015).

5 Bailey, P. *et al.* Genomic analyses identify molecular subtypes of pancreatic cancer. *Nature* **531**, 47-52 (2016).

6 Durinck, S., Spellman, P. T., Birney, E. & Huber, W. Mapping identifiers for the integration of genomic datasets with the R/Bioconductor package biomaRt. *Nat Protoc* **4**, 1184-1191, doi:10.1038/nprot.2009.97 (2009).

7 Network, C. G. A. R. Integrated Genomic Characterization of Pancreatic Ductal Adenocarcinoma. *Cancer Cell* **32**, 185-203 e113 (2017).

8 Maupin, K. A. *et al.* Glycogene expression alterations associated with pancreatic cancer epithelial-mesenchymal transition in complementary model systems. *PLoS One* **5**, e13002 (2010).

9 Barretina, J. *et al.* The Cancer Cell Line Encyclopedia enables predictive modelling of anticancer drug sensitivity. *Nature* **483**, 603-607 (2012).

10 Johnson, W. E., Li, C. & Rabinovic, A. Adjusting batch effects in microarray expression data using empirical Bayes methods. *Biostatistics* **8**, 118-127, doi:10.1093/biostatistics/kxj037 (2007).

11 Ritchie, M. E. *et al.* limma powers differential expression analyses for RNA-sequencing and microarray studies. *Nucleic acid Research* **43(7)**, 1362-4967 (2015).

12 Krijgsman, O., Kluin, R. & Peeper, D. XenofilteR. *GitHub*.

13 Garnett, M. J. *et al.* Systematic identification of genomic markers of drug sensitivity in cancer cells. *Nature* **483**, 570-575 (2012).
